# Influence of a Solid Surface on PNIPAM Microgel Films

**DOI:** 10.3390/gels10070473

**Published:** 2024-07-18

**Authors:** Valentina Nigro, Roberta Angelini, Elena Buratti, Claudia Colantonio, Rosaria D’Amato, Franco Dinelli, Silvia Franco, Francesca Limosani, Rosa Maria Montereali, Enrico Nichelatti, Massimo Piccinini, Maria Aurora Vincenti, Barbara Ruzicka

**Affiliations:** 1 ENEA C.R. Frascati, Nuclear Department, Via Enrico Fermi 45, 00044 Frascati, Italy; 2 Institute for Complex Systems, National Research Council (ISC-CNR), Sapienza University of Rome, P.le A. Moro 2, 00185 Rome, Italy; 3 Physics Department, Sapienza University, P.le Aldo Moro 2, 00185 Rome, Italy; 4 Department of Chemical, Pharmaceutical and Agricultural Sciences, University of Ferrara, Via Luigi Borsari 46, 14412 Ferrara, Italy; 5 National Institute of Optics (INO-CNR), Via Moruzzi 1, 56124 Pisa, Italy; 6 ENEA C.R. Casaccia, Nuclear Department, Via Anguillarese, 301, 00123 Rome, Italy

**Keywords:** microgels, PNIPAM, thin films, surface modification

## Abstract

Stimuli-responsive microgels have attracted great interest in recent years as building blocks for fabricating smart surfaces with many technological applications. In particular, PNIPAM microgels are promising candidates for creating thermo-responsive scaffolds to control cell growth and detachment via temperature stimuli. In this framework, understanding the influence of the solid substrate is critical for tailoring microgel coatings to specific applications. The surface modification of the substrate is a winning strategy used to manage microgel–substrate interactions. To control the spreading of microgel particles on a solid surface, glass substrates are coated with a PEI or an APTES layer to improve surface hydrophobicity and add positive charges on the interface. A systematic investigation of PNIPAM microgels spin-coated through a double-step deposition protocol on pristine glass and on functionalised glasses was performed by combining wettability measurements and Atomic Force Microscopy. The greater flattening of microgel particles on less hydrophilic substrates can be explained as a consequence of the reduced shielding of the water–substrate interactions that favors electrostatic interactions between microgels and the substrate. This approach allows the yielding of effective control on microgel coatings that will help to unlock new possibilities for their application in biomedical devices, sensors, or responsive surfaces.

## 1. Introduction

Stimuli-responsive microgels are soft colloidal particles made of crosslinked 3D polymer networks with unique properties [1,2], often used as model systems for investigating complex behaviors in soft matter and as building blocks for advanced technological applications in many different fields [3,4,5,6,7,8,9].

One of the most remarkable features of microgels is their sensitivity to external stimuli, such as temperature, pH and ionic strength [10,11,12], solvent [13,14,15], and composition [16,17,18,19]. In particular, polymeric microgels based on PNIPAM (poly(N-isopropylacrylamide)) deserve special attention since, due to the lower critical solution temperature (LCST) of the network-forming polymer, they exhibit a Volume Phase Transition (VPT) from a swollen to a collapsed state at a temperature of approximately 32 °C [14,20]. This behavior has been harnessed to fabricate smart materials with controlled drug release, sensing, and responsive surface properties [21,22]. Moreover, the intriguing behavior occurring near body temperature makes them especially attractive for biomedical and biotechnological applications [23,24,25].

When organized into films, microgels can arrange into unique microstructures such as layers, aggregates, or close-packed lattices [26,27,28]. Surface roughness, patterning, and nanostructuring are well-established methods for regulating the adhesion and arrangement of microgels at interfaces. Rough surfaces, specifically, provide extra anchoring points for microgels, which can lead to stronger physical or chemical interactions [29]. However, surface roughness can also impact the orientation and arrangement of microgels at the interface, potentially affecting their responsiveness. Moreover, patterned surfaces with defined geometries can direct the adhesion of microgels to specific regions, allowing precise control over their distribution on the substrate and enabling the fabrication of highly ordered microgel layers [30,31].

Over the past few decades, many deposition strategies have been employed to obtain polymeric films. Techniques such as spin-coating, dip coating, spray coating, and solvent evaporation are just a few examples of deposition methods used to fabricate highly functional microgel coatings [29,32,33,34]. Each of them offers distinct advantages and can be selected based on specific application requirements and substrate properties to produce versatile coatings. At the same time, these methods preserve the functionality and integrity of microgels, enabling scalability to larger substrates. In this context, spin-coating emerged as a very fast, inexpensive, and easily accessible technique that provides, at the same time, high control over the morphological properties of microgel films [35].

On the other hand, the responsiveness of microgels to external stimuli allows for adaptive changes in surface topography. In particular, thin films of PNIPAM microgels have been explored to develop responsive coatings with applications in various fields, including sensing [36,37], bio-sensing [21,38], and tissue engineering [39,40]. PNIPAM microgel films have been recently proposed as smart substrates to control cell growth and detachment via temperature stimuli to minimize the impact of enzymatic and chemical treatments generally used for cell detachment [39,41,42]. It is well known that cell adhesion and proliferation are favored by PNIPAM microgel properties above the Volume Phase Transition Temperature (VPTT), while switching back to room temperature results in repulsive forces allowing the removal of the cells from the substrate. Their biocompatibility and ability to mimic the extracellular matrix makes them suitable for creating scaffolds that support cell growth and tissue development. In this framework, parameters such as morphology, water content, elastic modulus, and adhesion are crucial parameters for controlling cell adhesion and detachment [40,43,44].

Understanding the influence of the solid substrate is critical for tailoring microgel coatings to specific applications. The interaction between microgels and the solid interface indeed plays a pivotal role in determining the coating’s adhesion, mechanical strength, and responsiveness to stimuli. For their potential use as switchable surfaces, obtaining uniform and stable microgel films is imperative. The surface modification of the substrate, such as the introduction of functional groups or coatings, can be employed to tailor the microgel–substrate interaction. On the other hand, this approach is essential to ensuring a stable and durable coating [42,45] or to retaining the spherical shape of the microgel particles [46]. Synthetic polymers are often employed to achieve the efficient and stable adsorption of PNIPAM microgels on the substrate [45,47]. Polyethylenimine (PEI), a cationic polyelectrolyte, is one of the most used synthetic polymers featuring a combination of primary, secondary, and tertiary amines. Low toxicity, high ionic charge density, easy separation, and the ability to chemically react with different functional groups make PEI especially attractive. Indeed, PEI has been extensively used as a substrate layer to ensure the adhesion of microgel particles. The high density of positive charges provided by the secondary and tertiary amines ensures the stable anchoring of PNIPAM microgels, offering a versatile method for designing surfaces with specific features. [48,49]. A different strategy is based on surface functionalization with aminopropyltriethoxysilane (APTES), one of the most studied organosilanes as a surface modifier. The moderate responsiveness of APTES makes it relatively easy to handle. The robust anchoring of the silane to the surface is ensured by its three hydrolysable ethoxy groups, while the amine function of the aminopropyl group remains available for further reactions [50,51]. It is well known that attractive interactions between the negatively charged microgels and the obtained positively charged substrates yield to the strong adsorption of the subchains, resulting in microgel spreading [52]. However, the ability of microgels to adhere to the substrate depends on the complex interplay between factors such as surface energy, roughness, chemical composition, and network elasticity. Optimizing these factors can enhance adhesion, ensuring a stable and durable coating. While surface modifications are commonly employed to enhance microgel adhesion on solid substrates, a comprehensive understanding of their impact on microgel behavior remains elusive. Specifically, the interactions between PEI and microgels are pivotal for numerous advanced applications in science and technology; however, a systematic investigation into the effects of PEI-modified surfaces on microgel coatings is still lacking. Conversely, pre-coating with APTES represents a relatively underexplored strategy that could prove particularly beneficial for creating cell culture surfaces. Typically, APTES acts as an adhesion promoter for PNIPAM chains, achieved either through a blended solution of PNIPAM and APTES or by forming an APTES network that immobilizes the PNIPAM layer on the surface. [53].

Here, we present a comprehensive study investigating how surface hydrophobicity influences microgel behavior at low and high weight concentrations. We utilized a double-step spin-coating protocol to precisely control microgel deposition on pristine glass as well as on glass surfaces functionalized with PEI or APTES layers. These surface modifications were employed to enhance surface hydrophobicity and introduce positive charges to the interface. Through a combination of wettability measurements and Atomic Force Microscopy (AFM), we examined how the solid substrate affects microgel shape and arrangement. Additionally, water contact angle measurements were conducted on densely packed films to explore how interactions between microgels and the substrate influence the thermoresponsive properties of the films.

## 2. Results and Discussion

The swelling behavior of PNIPAM microgels suspended in water was investigated through DLS at a high dilution limit (C_*w*_ = 0.01%), over a temperature range from 20 °C to 40 °C [35]. DLS measurements returned hydrodynamic diameter values at temperatures T = 20 °C and T = 40 °C of D_*H*_(20 °C) = (734 ± 61) nm and D_*H*_(40 °C) = (363 ± 19) nm, respectively. The swelling ratio α, defined as the ratio between the hydrodynamic diameters in the swollen and in the shrunken states, α = D_*H*_(20 °C)/D_*H*_(40 °C), was α ≈ 2.0, indicating a high shrinking capability. Indeed, a relatively low crosslinker-to-monomer ratio (BIS/NIPAM) of 1.3 mol% was chosen, as described in the synthesis procedure, to achieve soft and deformable particles, in order to obtain densely packed films with a uniform and continuous coverage of the substrate.

**Figure 1:**
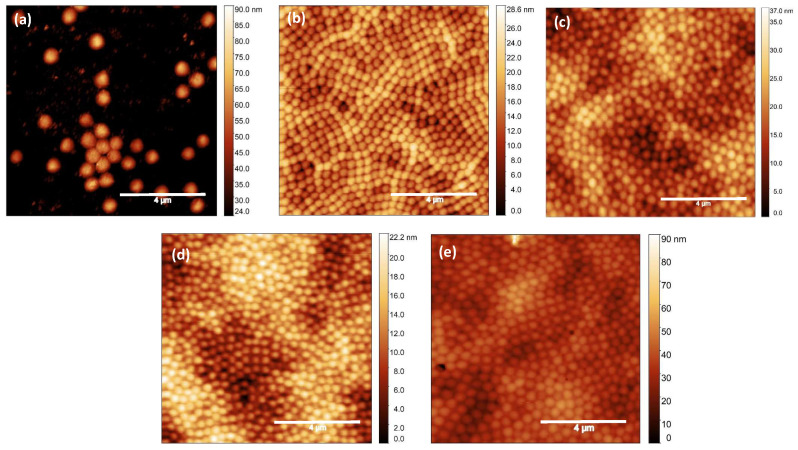
AFM images over a 10 × 10 μm^2^ area of PNIPAM micrrogels spin-coated on pristine glass substrates at concentrations of (**a**) C_*w*_ = 0.1%, (**b**) C_*w*_ = 0.5%, (**c**) C_*w*_ = 1.0%, (**d**) C_*w*_ = 3.0%, and (**e**) C_*w*_ = 5.0%. Scale bar: 4 μm.

A volume V = 50 μL of aqueous suspensions of PNIPAM microgel at five different concentrations (C_*w*_ = 0.1%, C_*w*_ = 0.5%, C_*w*_ = 1.0%, C_*w*_ = 3.0%, and C_*w*_ = 5.0%) were spin-coated on pristine glass without previous treatment to investigate the effect of microgel concentration on their shape and arrangement when deposited on a solid substrate. A double-step deposition protocol with low rotation speed in the first step ($\omega_{1}$ = 500 rpm) and a high rotation speed in the second step ($\omega_{2}$ = 5000 rpm) was used [35]. Figure 1 shows AFM images over a 10 × 10 μm^2^ area for microgels at different weight concentrations spin-coated on pristine glass. At a low microgel concentration (C_*w*_ = 0.1%), particles are well separated, and clustering is avoided. This allowed us to measure the single-microgel diameter, which was found to be D_*AFM*_ = (571 ± 18) nm. This result indicates a broadening of the microgel particle on the substrate by more than 60% with respect to their hydrodynamic diameter in the shrunken state. With increasing concentration (C_*w*_ = 0.5–5%), the microgel lateral size decreases up to about 250 nm as a consequence of the lateral compression induced by neighboring particles. However, regardless of the concentration, all microgel particles show heights of the order of tens of nanometers, indicating a high transversal compression as expected for soft microgels at a low crosslinker concentration. Moreover, homogeneous and smooth films are formed with microgels arranging in the typical hexagonal close-packed structure. However, a monolayer configuration seems to be obtained only at C_*w*_ = 0.5%, while at higher weight concentration values, compact and smooth multilayer structures were obtained that could be exploited to ensure an optimized coverage degree of the substrate.

**Figure 2:**
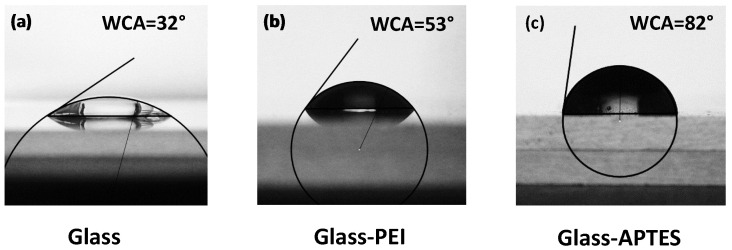
Water contact angle (WCA) measurements on (**a**) pristine glass, (**b**) glass modified with PEI, and (**c**) glass modified with APTES.

However, microgels may exhibit different behaviors on hydrophobic or hydrophilic surfaces since the substrate wettability is known to affect their spreading and adhesion. Tailoring the surface energy of the substrate to match the microgel properties can improve wetting and enhance the overall coating performances. To control the spreading behavior of the microgel particles on a solid surface, two different surface modifications were used. The glass substrate was coated with a PEI or an APTES layer to improve surface hydrophobicity and add positive charges on the interface. The modifications were verified via contact angle measurements: a water droplet of V = 10 μL was deposited on pristine glass and on glass functionalized with PEI and APTES, resulting in a water contact angle (WCA) of 34°, 53°, and 82°, respectively. Results from the contact angle measurements are shown in Figure 2 and indicate more hydrophobic surfaces for functionalized glasses than for pristine glass.

**Figure 3:**
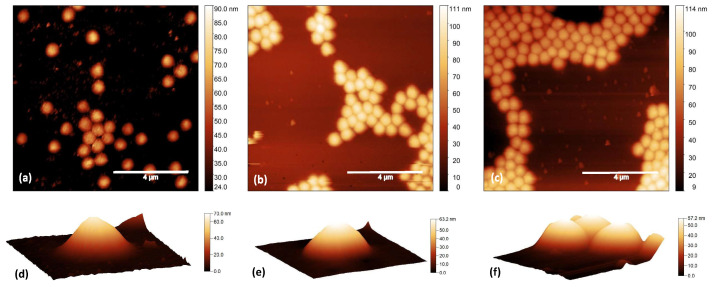
AFM images over a 10 × 10 μm^2^ area of PNIPAM microgels at C_*w*_ = 0.1% spin-coated on (**a**) pristine glass, (**b**) glass functionalized with PEI, and (**c**) glass functionalized with APTES. Three-dimensional images of mostly isolated particles on (**d**) pristine glass, (**e**) glass functionalized with PEI, and (**f**) glass functionalized with APTES. Scale bar: 4 μm.

To investigate the effect of surface modifications on the microgel shape and arrangement, highly diluted microgel suspensions (C_*w*_ = 0.1%) were spin-coated on pristine glass and on both glass–PEI and glass–APTES substrates. It is well known that relatively soft microgels adsorbed on solid surfaces may assume a non-spherical structure that can be controlled by the surface hydrophobicity. Indeed, the spreading and adhesion of microgels on the substrate is driven by the shielding of weak water–substrate interactions and is therefore expected to increase at increasing hydrophobicity [46,52]. Moreover, attractive interactions between the soft particles and the substrates cause the strong adsorption of the subchains, further increasing microgel spreading. In our case, the anchoring of microgels onto the glass surface is greatly enhanced because of the electrostatic attraction between negatively charged microgels and positvely charged glass–PEI or glass–APTES surfaces. AFM images acquired over a 10 × 10 μm^2^ area of PNIPAM microgels at C_*w*_ = 0.1% spin-coated on pristine glass and on glasses functionalized with PEI or APTES are reported in Figure 3 together with 3D images of mostly isolated particles to better visualize their shape and flattening. Values for the particle height, Root Mean Square (RMS) roughness, and lateral particle size obtained from the AFM images for microgel films spin-coated on different substrates are reported in Table 1, together with the substrate WCA values.

Interestingly, particle height slightly decreases on glass–PEI and glass–APTES with respect to pristine glass, while particle lateral size increases. This confirms the greater flattening of microgel particles on more hydrophobic surfaces as a consequence of the reduced shielding of water–substrate interactions. Moreover, surface modification induced a slight increase in the Root Mean Square (RMS) roughness from the 1.3 of pristine glass up to ≈2 nm for glass substrates coated with PEI or APTES. This higher surface roughness might provide more binding sites for the microgel dangling ends, which probably leads to a higher surface packing favoring their aggregation in larger clusters.

**Figure 4:**
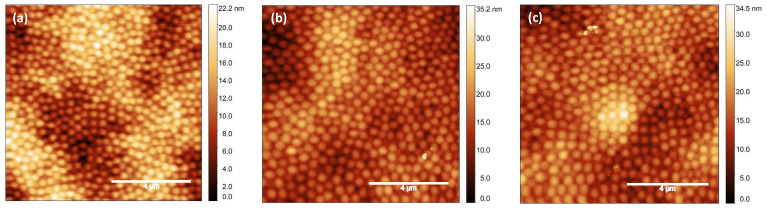
AFM images of PNIPAM microgels at C_*w*_ = 3.0% spin-coated on (**a**) pristine glass, (**b**) glass functionalized with PEI, and (**c**) glass functionalized with APTES. Scale bar: 4 μm.

These results suggest that the strength of the interaction between microgels and solid substrates may control the shape and flatting of microgels, which is in turn expected to control the texture of a close-packed film. In this perspective, microgel suspensions at a high weight concentration (C_*w*_ = 3.0%) were spin-coated through the same deposition protocol on pristine glass and on glass–PEI and glass–APTES substrates. From the AFM images reported in Figure 4, it is confirmed that this high concentration supports the formation of close-packed films on different surfaces. Moreover, microgel films at C_*w*_ = 3.0% spin-coated on glass–PEI and glass–APTES substrates are characterized by an RMS roughness of approximately 5 nm, in contrast to the higher RMS roughness of approximately 15 nm observed in films spin-coated on pristine glass. This suggests that the flattening of microgels on less hydrophilic substrates favors particle overlapping and therefore the formation of highly packed and uniform coatings.

**Figure 5:**
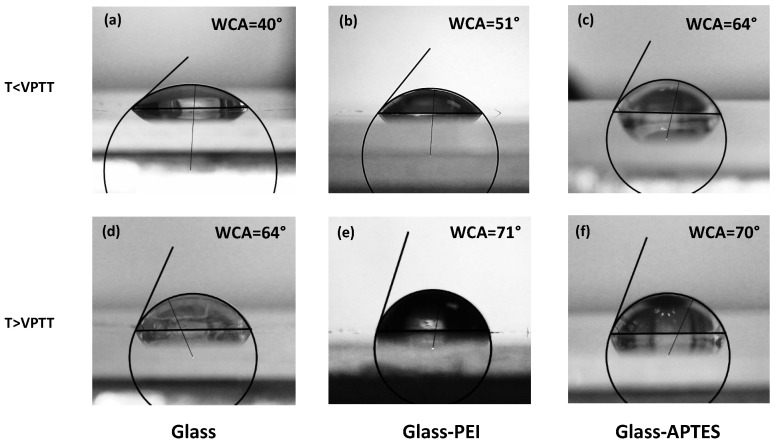
Water contact angles measured on PNIPAM microgel films at C_*w*_ = 3%, at temperatures below the microgel VPTT, spin-coated on (**a**) pristine glass, (**b**) glass modified with PEI, and (**c**) glass modified with APTES and at temperatures above the microgel VPTT measured on the same films on (**d**) pristine glass, (**e**) glass modified with PEI, and (**f**) glass modified with APTES.

However, for their application as smart coatings, the influence of a solid substrate on the microgel film thermoresponsiveness must be taken into account. Indeed, the hydrophilic-to-hydrophobic transition of microgel particles could be hugely affected by the presence of a solid surface. To test their response to temperature changes, a water droplet of volume V = 10 μL was deposited on microgel films at C_*w*_ = 3.0% spin-coated on pristine glass, glass–PEI, and glass–APTES at a temperature below and above the VPTT (Figure 5). Interestingly, the hydrophilic character of microgel films at a temperature below the VPTT is slightly affected by surface modifications. From the water contact angle measurements, PNIPAM microgel films spin-coated on pristine glass were found to be rather hydrophilic, while higher WCA values were obtained if the microgels were spin-coated on more hydrophobic substrates. In increasing the temperature above the VPTT, the contact angle behavior shows an increment in the hydrophobicity surface due to the more hydrophobic character of PNIPAM microgels in the shrunken state. However, the transition from a hydrophilic to a more hydrophobic state is moderately reduced in films deposited on glass–PEI and glass–APTES as a consequence of the stronger attractive interaction between the slightly negatively charged microgels and the positively charged surfaces.

## 3. Conclusions

To explore the effects of solid surfaces on microgel shape and arrangement, PNIPAM microgels were spin-coated at different weight concentrations on pristine glass and on glass pre-coated with a PEI or an APTES layer to increase substrate hydrophobicity and add positive charges on the surface. A systematic investigation of film properties was performed by combining wettability measurements and Atomic Force Microscopy. At a low weight concentration, microgel particles are well separated and flattened onto the surfaces. However, with increasing hydrophobicity of the substrate, greater spreading and adhesion of microgel particles are observed. This can be explained as a consequence of the reduced shielding of weak water–substrate interactions, which favors attractive electrostatic interactions between negatively charged microgels and positevely charged surfaces. At a higher weight concentration, uniform and smooth thin films were obtained both on pristine glass and on glass–PEI or glass–APTES by following a double-step spin-coating deposition protocol previously optimized to ensure a high degree of substrate coverage, which may have dramatic effects on their application as smart coatings. Moreover, water contact angle measurements were performed on highly packed films at temperatures below and above the VPTT to investigate the influence of microgel–substrate interactions on film thermoresponsiveness. At T < 32°, a slight reduction in the hydrophilic character of microgel films spin-coated on glass–PEI and glass–APTES was observed due to the stronger attractive interactions between microgels and the solid substrate. On the other hand, the transition to an hydrophobic state at T > 32° is not significantly affected by surface modification, thus confirming the possibility to exploit the typical hydrophilic-to-hydrophobic transition of PNIPAM microgels for their application as switchable coatings. This approach paves the way to their use as promising tools to assemble tissue-engineered microenvironments, providing excellent control over cell adhesion, proliferation, and detachment.

## 4. Materials and Methods

### 4.1. Materials

N-isopropylacrylamide (NIPAM) (monomer), N,N′-methylene-bis-acrylamide (BIS) (crosslinker), potassium persulfate (KPS) (98% purity) (initiator), and poly(ethyleneimine) (PEI) solution (average Mw 750,000, 50 wt% in H_2_O), (3-Aminopropyl)triethoxysilane (APTES) (≤98% purity) were purchased from Merck KGaA (Darmstadt, Germany). NIPAM and BIS were recrystallized from hexane and methanol, respectively, dried under reduced pressure (0.01 mmHg) at room temperature and stored at 253 K. All the other reagents and solvents, purchased from Merck KGaA (Darmstadt, Germany), were used as received. Ultrapure water, with resistivity 18.2 MΩ/cm at 25 °C, was obtained through the Sarium^®^ pro Ultrapure water purification Systems, Sartorius Stedim. Prior to use, dialysis membrane, SpectraPor^®^ 1, MWCO 6–8 kDa (Spectrum Laboratories, Inc., Piscataway, NJ, USA) was soaked in distilled water for 2 h and then thoroughly rinsed.

### 4.2. Microgel Synthesis

To synthesize PNIPAM microgels, surfactant-free precipitation polymerization was used. In particular, 3.716 g of NIPAM and 0.065 g of BIS were solubilized in 230 mL of ultrapure water and transferred into a 250 mL four-necked jacketed reactor equipped with a condenser and a mechanical stirrer. The molar ratio of BIS to NIPAM was set at 1.3 mol% to ensure the formation of deformable particles. Nitrogen was purged for one hour to deoxygenate the solution, successively heated at 70 °C. The polymerization was initiated by adding 0.161 g of KPS dissolved in 10 mL of deoxygenated water, and the reaction was allowed to proceed for 4 h. The obtained PNIPAM microgel was purified via dialysis with a MWCO 6–8 kDa membrane against distilled water with frequent water replacements over two weeks. Gravimetric analysis determined the final weight concentration (C_*w*_) of the PNIPAM microgel to be approximately 1%. To obtain samples with higher concentrations of 10 wt%, the microgel was lyophilized to dryness and then redispersed in H_2_O. Samples at different concentrations were obtained by diluting the 10 wt% solution.

### 4.3. Surface Modification

**Scheme 1:**
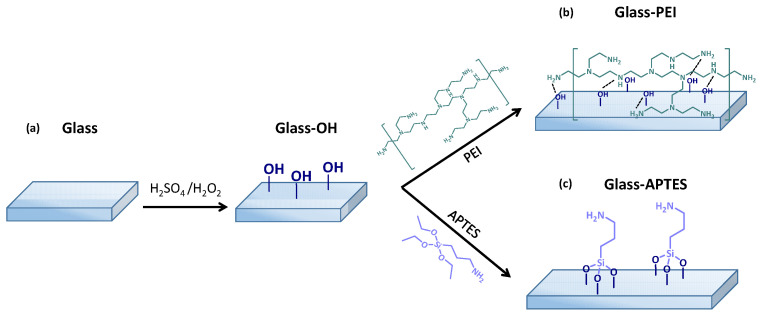
Scheme of surface modifications of (**a**) pristine glass to obtain (**b**) glass–PEI and (**c**) glass–APTES substrates.

Glass slides (25 mm × 25 mm) were ultrasonically washed with water and ethanol with no previous treatment (Scheme 1a). Freshly prepared piranha solution (70 vol.% of concentrated H_2_SO_4_ and 30 vol.% of 30% H_2_O_2_) was used to remove organic contaminants from the glass surface and obtain a glass–OH surface. Before surface modification, the glass slides were immersed in the piranha solution for 5 min at 120 °C and then thoroughly rinsed with deionized water and dried with ultrapure air. To obtain glass–PEI (Scheme 1b) and glass–APTES (Scheme 1c) surfaces, the glass–OH surfaces were treated with a PEI or APTES aqueous solution (1 wt% PEI and 10 wt% APTES) for 2 h at 70 °C. Thereafter, the treated substrates were washed with EtOH and dried with a ultrapure air flow. Substrates were stored in a desiccator until use.

### 4.4. Thin-Film Deposition

Thin films of PNIPAM microgels were obtained through a standard spin-coater Polos SPIN150i/200i infinite (SPS-EUROPE B.V., Putten, The Netherlands) through a double-step deposition protocol with the first step spin speed ($\omega_{1}$) set at 500 rpm and the second step spin speed ($\omega_{2}$) of 5000 rpm, previously optimized by our group [35]. In total, 50 μL of aqueous suspensions of PNIPAM microgels at five different weight concentrations (C_*w*_ = 0.1%, 0.5%, 1.0%, 3.0%, 5.0%) were spin-coated on pristine glass substrates. Two weight concentrations, namely C_*w*_ = 0.1% and 3%, were selected for microgel deposition on glass substrates functionalized with PEI or APTES.

### 4.5. Characterization

#### 4.5.1. Dynamic Light Scattering

A single-angle setup was used to perform DLS measurements in the time range between $10^{-6}$ s and 1 s. A solid state laser with wavelength of 632.8 nm (power of 10 mW) and single-mode collecting fiber at the scattering angle θ = 90° were used. Time autocorrelation functions were therefore obtained by calculating the intensity autocorrelation function g_2_(Q,t) = $\frac{\langle I(Q,0)I(Q,t)\rangle }{{\langle I(Q,0)\rangle }^{2}}$ at the scattering vectors Q = (4πn/λ) sin(θ/2). Intensity autocorrelation functions were fitted through the Kohlrausch–Williams–Watts expression [54,55] g_2_(Q,t) = $1+b[{\left(e^{-{\left(t/\tau \right)}^{\beta }}\right]}^{2}$, where *b* is the coherence factor, τ is an “effective” relaxation time, and β describes the deviation from the simple exponential decay (β = 1). In particular, the β parameter gives a measure of the relaxation time distribution due to the intrinsic sample polydispersity, and it was found to be β ≈ 1 at low concentrations at all investigated temperatures, thus indicating very monodisperse samples. DLS measurements were performed in the temperature range T = (20–40) °C at a low weight concentration (C_*w*_ = 0.01%) to obtain the hydrodynamic diameters.

#### 4.5.2. Atomic Force Microscopy

For the investigation of the morphological properties of both substrates and films, AFM measurements were performed using the A100 PLUS AFM (A.P.E. Research s.r.l., Area Science Park, Basovizza (TS), Italy) and a hybrid system made of a commercial head NT-MDT SMENA (Spectrum Instruments, Limerick, Ireland), home-built electronics, and a digital lock-in amplifier Zurich HF2LI (Zurich Instruments, Zurich, Switzerland), operating in non-contact mode. All images were taken in air-ambient conditions, with commercial MikroMasch cantilevers (MikroMasch Europe, Wetzlar, Germany) HQ:NSC15 (nominal force constant from 20 to 80 N/m and resonance frequency from 265 to 410 kHz) and HQ:NSC35 (nominal force constant from 5.4 to 16 N/m and resonance frequency from 130 to 300 kHz). The open-source software Gwyddion (Gwyddion 2.65, Czech Metrology Institute, Brno, Czech Republic) was used for topography and statistical analyses [56].

#### 4.5.3. Contact Angle Measurements

For investigating the wetting properties of the glass surfaces, a specific in-house optical setup for contact angle measurements was employed. The setup consists of a fiber illuminator that backlights the sample through a diffuser, an adjustable sample holder on which the substrate is placed, and a portable Dino-Lite AM4515ZT digital microscope [57] connected to a computer with the dedicated software DinoCapture 2.0. This setup allows for the visualization of the magnified droplet image and facilitates the analysis for contact angle measurements. Contact angle measurements were performed at two different temperatures, below and above the microgel VPTT, to investigate the hydrophilic/hydrophobic nature of the PNIPAM microgel films on functionalized glass substrates.

## Figures and Tables

**Table 1:** Water contact angles (WCAs) and statistical quantities obtained from AFM images for thin films of PNIPAM microgels at C_*w*_ = 0.1% spin-coated on pristine glass, glass–PEI, and glass–APTES.

Substrate	WCA	Particle Height (nm)	RMS Roughness (nm)	Size (nm)
Glass	32°	70 ± 1	9.2 ± 0.2	571 ± 18
Glass–PEI	53°	63 ± 3	19 ± 2	633 ± 15
Glass–APTES	82°	57 ± 3	20 ± 2	662 ± 11

## Data Availability

The original contributions presented in the study are included in the article, further inquiries can be directed to the corresponding author.
